# Impact and Possible Mechanism(s) of Adipose Tissue-Derived Mesenchymal Stem Cells on T-Cell Proliferation in Patients With Rheumatic Disease

**DOI:** 10.3389/fphys.2021.749481

**Published:** 2022-01-13

**Authors:** Ewa Kuca-Warnawin, Marzena Olesińska, Piotr Szczȩsny, Ewa Kontny

**Affiliations:** ^1^Department of Pathophysiology and Immunology, National Institute of Geriatrics, Rheumatology and Rehabilitation, Warsaw, Poland; ^2^Clinic of Connective Tissue Diseases, National Institute of Geriatrics, Rheumatology and Rehabilitation, Warsaw, Poland

**Keywords:** systemic lupus erythematosus, systemic sclerosis, adipose-derived mesenchymal stem cells, T-cell proliferation, soluble mediators

## Abstract

**Objectives:** Systemic lupus erythematosus (SLE) and systemic sclerosis (SSc) are chronic wasting, incurable rheumatic diseases of autoimmune background, in which T cells play a critical pathogenic role. Autologous adipose tissue-derived mesenchymal stem cells (ASCs) may represent an alternative therapeutic option for SLE and SSc patients, but the biology of these cells is poorly understood.

**Methods:** Herein, we evaluated the anti-proliferative impact of ASCs of healthy donors (HD/ASCs, 5 reference cell lines), SLE patients (*n* = 20), and SSc patients (*n* = 20) on T lymphocytes. To assess the direct and indirect pathway of ASCs action, peripheral blood mononuclear cells (PBMCs) and purified CD4+ T cells of HD were activated and co-cultured in cell-to-cell contact (C-C) and transwell (T-W) conditions with untreated or cytokine (TNF + IFNΥ, TI)-licensed ASCs, then analyzed by flow cytometry to rate the proliferation response of CD8^+^ and/or CD4^+^ T cells. The concentrations of kynurenines, prostaglandin E2 (PGE_2_), interleukin 10 (IL-10), and transforming growth factor β (TGFβ) were measured from culture supernatants. Specific inhibitors of these factors (1-MT, indomethacin, and cytokine-neutralizing antibody) were used to assess their contribution to anti-proliferative ASCs action.

**Results:** All tested ASCs significantly decreased the number of proliferating CD4^+^ and CD8^+^ T cells, the number of division/proliferating cell (PI), and fold expansion (RI), and similarly upregulated kynurenines and PGE_2_, but not cytokine levels, in the co-cultures with both types of target cells. However, TI-treated SLE/ASCs and SSc/ASCs exerted a slightly weaker inhibitory effect on CD4^+^ T-cell replication than their respective HD/ASCs. All ASCs acted mainly *via* soluble factors. Their anti-proliferative effect was stronger, and kynurenine levels were higher in the T-W condition than the C-C condition. Blocking experiments indicated an involvement of kynurenine pathway in inhibiting the number of proliferating cells, PI, and RI values as well as PGE_2_ role in decreasing the number of proliferating cells. TGFβ did not contribute to ASCs anti-proliferative capabilities, while IL-10 seems to be involved in such activity of only SLE/ASCs.

**Conclusion:** The results indicate that SLE/ASCs and SSc/ASCs retain their capability to restrain the expansion of allogeneic CD4^+^ and CD8^+^ T cells and act by similar mechanisms as ASCs of healthy donors and thus may have therapeutic value.

## Introduction

Systemic lupus erythematosus (SLE) and systemic sclerosis (SSc) are devastating and potentially fatal rheumatic diseases of an autoimmune background. Both diseases are characterized by activation and dysregulation of T, B, and other immune cells; production of autoantibodies of various specificities; chronic inflammation; and life-threatening complications (Tsokos, 2011; Denton and Khanna, 2017). In SLE, the multiple pathogenic processes finally lead to the damage of numerous target organs, e.g., skin, kidney, joints, and brain (Tsokos, 2020). In SSc, microvascular abnormalities, inadequate immune response, and chronic inflammation, all contribute to progressive fibrosis of the skin and internal organs (Varga et al., 2017).

T lymphocytes play a central role in the establishment and maintenance of self-tolerance to protect against autoimmunity. However, in SLE and SSc, various T-cell subsets play a critical pathogenic role by generating and maintaining the autoimmune response, providing help to B-cells, amplifying systemic inflammation, and contributing to tissue and organ damage (Moulton et al., 2017; Varga et al., 2017; Asano and Varga, 2020; Tsai et al., 2020; Tsokos, 2020; Chen and Tsokos, 2021). The presence of both CD4^+^ and CD8^+^ T cells in the affected tissues was confirmed in SLE patients (Li et al., 2007; Cohen et al., 2008; Crispin et al., 2008) and SSc patients (Almeida et al., 2015; Liu et al., 2016; Fuschiotti, 2018a,b). To execute their effector functions, T cells are critically dependent on cellular proliferation. The excessive proliferation of CD4^+^, CD8^+^, and double-negative (CD4^–^CD8^–^) T lymphocytes was observed in murine models of lupus and SLE patients (Tang et al., 2009; Balomenos et al., 2017; Du et al., 2019), while increased proliferation rate of naive and functionally differentiated CD4^+^ T cells was detected in SSc patients (Giovanetti et al., 2010).

Despite advances in therapeutic opportunities, SLE and SSc are still incurable diseases. Even though the conventional immunosuppressive drugs exert curative effects in some SLE patients, severe side effects and refractoriness to these drugs make them ineffective in others (Tsokos, 2011). In SSc, current therapies are only symptomatic and help to alleviate clinical manifestation and disease-related pathologies (Asano and Varga, 2020). Until now, the application of biological agents in SLE and SSc is also discouraging (Magro, 2019; Asano and Varga, 2020). Thus, there is a need for more beneficial and safer treatment for both diseases.

It is well documented that mesenchymal stem/stromal cells (MSCs), residing in various tissues, have capabilities to effectively modulate immune response (Shi et al., 2018; Müller et al., 2021). Unfortunately, bone marrow-derived MSCs (BM-MSCs) from SLE patients exhibit functional deficiencies, e.g., senescent phenotype, impaired migration, differentiation, and immune regulation (El-Jawhari et al., 2021; Li et al., 2021; Tang et al., 2021), while adipose tissue-derived MSCs (ASCs) are poorly characterized (Kuca-Warnawin et al., 2019, 2020). Therefore, in ongoing clinical trials, aimed at patients with severe and refractory SLE, allogeneic MSCs are transplanted (Cheng et al., 2019; El-Jawhari et al., 2021; Li et al., 2021; Tang et al., 2021). Accumulating evidence indicates, however, that MSCs are not immune privileged and elicit immune response in allogeneic recipient (Ankrum et al., 2014). In SSc, both BM-MSCs and ASCs may contribute to disease pathology through the adipocyte-to-myofibroblasts transition process, but there is controversy over their immunomodulatory potential (Maria et al., 2017; Rosa et al., 2021). Nevertheless, preclinical and clinical studies have reported good therapeutic effects of autologous ASCs-based local treatment of SSc patients for cutaneous hand and facial manifestations (Rosa et al., 2021). In SLE and SSc chronic inflammation, the characteristics of autoimmune diseases are thought to be responsible for the abnormal functions of MSCs. Because in these types of diseases the inflammatory response in the bone marrow and inflamed fat pad is of higher grade than that in the peripheral adipose tissue (Kontny and Prochorec-Sobieszek, 2013; Kurowska and Kuca-Warnawin, 2016), it is likely that biological functions of ASCs from subcutaneous fat are better preserved than BM-MSCs. Furthermore, accumulating data suggest the superiority of ASCs over BM-MSCs in regulating inflammation, since ASCs show lower transcriptome heterogeneity and higher immunosuppressive capacity (Metlief et al., 2013; Zhou et al., 2019; Ménard et al., 2020).

Thus, in terms of further systemic therapeutic application of autologous ASCs in SLE and SSc patients, it is important to definitely find out whether these cells have similar immunosuppressive properties to ASCs of healthy donors. For this reason, we have assessed the anti-proliferative effects of ASCs, isolated from subcutaneous fat of SLE (SLE/ASCs) and SSc (SSc/ASCs) patients, on CD4^+^ and CD8^+^ T cells using ASCs originating from healthy donors (HD/ASCs) as the reference cell lines. To this aim, activated target cells were co-cultured with untreated or pro-inflammatory cytokine-licensed ASCs and then their inhibitory effect on T-cell proliferation was assessed; the contribution of several soluble mediators, i.e., kynurenines, prostaglandin E_2_ (PGE_2_), transforming growth factor β (TGFβ), and interleukin (IL) 10, to ASCs anti-proliferative effect was verified by the application of co-cultures preventing direct cell-to-cell contact and by using specific inhibitors of these factors. As MSCs are known to inhibit T-cell functions both directly (Yañez et al., 2010; Li et al., 2014) and indirectly *via* engagement of intermediary cells—mostly monocytes (Cutler et al., 2010; Wang et al., 2010), we also assessed the functionality of both ways of ASCs action using purified CD4^+^ T cells and peripheral blood mononuclear cells (PBMCs) as the target cells.

## Materials and Methods

### Patients and Sample Collection

Two groups of patients, who fulfilled the criteria for SLE (*n* = 20) (Petri and Orbai, 2012) or SSc (*n* = 20) (Van den Hoogen and Khanna, 2013), were included in the study (Table 1). This study meets all criteria contained in the Declaration of Helsinki and was approved by the Ethics Committee of the National Institute of Geriatrics, Rheumatology and Rehabilitation, Warsaw, Poland (the approval protocol no: KBT-8/4/20016). Before enrolment, all patients gave their written informed consent.

**TABLE 1:** Demographic and clinical characteristics of the patients.

	Systemic lupus erythematosus (SLE)(*n* = 20)	Systemic sclerosis (SSc)(*n* = 20)
**Demographics**		
Age, years	43 (25)	49 (17)
Sex, female (F) / male (M), n	19F/1M	14F/6M
BMI	24.5 (13.3)	24.4 (3.4)
Disease duration, years	8 (15)	5 (8)^a^/3 (5.5)^b^
**Clinical data**		
Disease activity, score	6 (12)*	1 (1.9)**
**Laboratory values**		
CRP, mg/L	5 (9.5)	7 (5.5)
ESR, mm/h	17 (18)	17 (15)
Proteinuria, mg/24 h	65 (398)^#^	0 (0.07)^#^
C3, mg/dL	80.1 (41.9)	97.1 (24.75)
C4, mg/dL	15.5 (12.17)	16.6 (4.75)
ANA, titer (1:x)	1270 (3200)^##^	5120 (5120)^##^
anti-dsDNA antibody, %	84.6	n/a
anti-dsDNA antibody, IU/mL	54.6 (220)	n/a
Scl-70 antibody, %	n/a	64.7
Autoantibody specificities, no.,	4 (2)^##^	3 (1)^##^
Medications, %		
Immunosuppressive drugs	100	100

*Except where indicated otherwise, values are the median (IQR).*

*BMI, body mass index; duration of ^a^Raynaud’s syndrome or ^b^skin/organ symptoms; CRP, C-reactive protein; ESR, erythrocyte sedimentation rate; C, complement components; ANA, antinuclear antibody; Scl-70, anti-topoisomerase I antibody; n/a, not applicable.*

*SLEDAI, SLE Disease Activity Index, **EUSTAR, the European League against Rheumatism Scleroderma Trials and Research revised index.

*^#^P = 0.05–0.01 and **^##^**P = 0.01–0.001 for SLE versus SSc comparisons.*

### Adipose Tissue-Derived Mesenchymal Stem Cells Isolation and Culture

Specimens of subcutaneous abdominal fat were taken from the patients by 18 G needle biopsy. Tissue processing, ASCs isolation, and culture were performed as described previously (Skalska and Kontny, 2012). Five human adipose-derived mesenchymal cell lines from healthy volunteers (Lonza Group, Lonza Walkershille Inc., MD, United States; donor numbers: 0000440549, 0000410252, 0000535975, 0000605220, 0000550179) were used as a control. All experiments were performed using ASCs at 3–5 passages. ASCs were cultured in a complete culture medium composed of DMEM/F12 (PAN Biotech United Kingdom Ltd., Wimborn, United Kingdom), 10% fetal calf serum (FCS) (Biochrom, Berlin, Germany), 200 U/ml penicillin, 200 μg/ml streptomycin (Polfa Tarchomin S.A., Warsaw, Poland), and 5 μg/ml plasmocin (InvivoGen, San Diego, CA, United States). Both untreated and cytokine-licensed ASCs were applied. To this aim, ASCs were stimulated for 24 h with human recombinant tumor necrosis factor (TNF) and interferon γ (IFNγ) (both from R&D Systems, Minneapolis, MN, United States; each applied at 10 ng/ml) (ASCs_*TI*_).

### Co-cultures of Adipose Tissue-Derived Mesenchymal Stem Cells With Allogeneic Target Cells

All co-cultures were performed in the complete DMEM/F12 medium. ASCs (6 × 10^4^/well/2 ml of medium) were seeded into 24-well plates and stimulated with IFNγ and TNF (see above). PBMCs were isolated from buffy coats obtained from healthy male honorary blood donors (<60 years old), according to routinely applied procedure with the use of Ficoll-Paque (GE Healthcare, Uppsala, Sweden). The CD3^+^CD4^+^ cells were isolated from PBMCs using EasySep™ Human CD4^+^ T Cell Isolation Kit (Stemcell Technologies, Vancouver, Canada). After isolation, PBMCs or CD4^+^ T cells (1.2 × 10^6^/well/2 ml of medium) were seeded either directly (contacting co-culture) or on a 0.4 μm pore size Transwell filters (MD24 with a carrier for inserts 0.4 MY; Thermo Fisher Scientific, Massachusetts, MA, United States) (non-contacting co-culture) into 24-well plates with 6 × 10^4^/well or 5 × 10^4^/well adherent ASCs, respectively. Then, PBMCs were treated with 2.5 μg/ml of phytohemagglutinin (PHA; Sigma-Aldrich, St. Louis, MO, United States), while T cells were activated with Dynabeads™ Human T-Activator CD3/CD28 (Thermo Fisher Scientific, Massachusetts, MA, United States) or with PHA in some experiments. After 5 days of co-culture, culture supernatants and target cells were harvested for further analysis, i.e., the measurement of kynurenines, PGE_2_, IL-10, and TGFβ concentrations, or flow cytometry, respectively. PBMCs or CD4^+^ T cells cultured separately were used as a control.

### Measurement of Soluble Factors Concentrations

Kynurenine concentration was measured spectrophotometrically as described previously (Kuca-Warnawin et al., 2019). The optical density of the samples was measured at a wavelength of 490 nm. L-kynurenine (Sigma-Aldrich, St. Louis, MO, United States) diluted in culture medium was used to prepare the standard curve. The concentration of PGE_2_ was measured using the Parameter Kit (R&D Systems, Minneapolis, MN), while cytokine concentrations were measured using specific enzyme-linked immunosorbent assays (ELISAs), i.e., TGFb ELISA DuoSet Kits from R&D Systems and IL-10 ELISA (cat. no. 88-7104-88) from Invitrogen (Vienna, Austria). All measurements were done in duplicates.

### Flow Cytometry

Peripheral blood mononuclear cells harvested from cultures were resuspended in 50 μl of FACS buffer and stained for 30 min on ice for respective membrane antigens using fluorochrome-conjugated monoclonal antibodies specific for human: CD3-FITC, CD4-APC-Cy7 (both obtained from BD Pharmingen, San Diego, CA, United States), or CD8-PerCP (eBioscience, San Diego, CA, United States). After the washing step, cells were acquired and analyzed using a FACSCanto cell cytometer and Diva software. Appropriate isotype controls were used in all experiments. For proliferation assay, PBMCs and purified CD4^+^ T cells were stained with cell trace violet (CTV) (Thermo Fisher Scientific, Massachusetts, MA, United States) and then stimulated and co-cultured with ASCs as described above. Cells harvested from cultures were analyzed by flow cytometry to identify proliferating and non-proliferating cells. To characterize cellular proliferation response, the percentage of proliferating cells, proliferation index (PI), and replication index (RI) were calculated as described elsewhere (Roederer, 2011), using the following mathematical formulas:


$$\text{PI}=\frac{\sum_{1}^{i}i\times \frac{N_{i}}{2^{i}}}{\sum_{1}^{i}\frac{N_{i}}{2^{i}}}$$

Proliferation index—for responding cells, an average number of a division they have undergone; *N* = number of cells in the division; *i* = number of division:


$$\text{RI}=\frac{\sum_{1}^{i}N_{i}}{\sum_{1}^{i}\frac{N_{i}}{2^{i}}}$$

Replication index—for responding cells, fold expansion over the culture time; *N* = number of cells in the division; *i* = number of division.

### Blocking Experiments

To investigate the role of soluble factors in immunomodulatory capacity of ASCs, specific inhibitors of PGE_2_ and kynurenines synthesis, i.e., 1 mM of indomethacin (Sigma Aldrich, Germany), 1 mM of 1-methyltryptophan (1-MT, Sigma Aldrich, Germany), 50 μg/ml TGFβ-neutralizing antibody (1D11.16.8), or 5 μg/ml IL-10-neutralizing antibody (JES3-9D7) (both from Thermo Fisher Scientific, Massachusetts, MA, United States), were added at the beginning of the cell culture periods. The above concentrations were selected from previous studies (Kuca-Warnawin et al., 2021a). After 48 h, specific inhibitors and neutralizing antibodies were added again. Next, PHA-activated and CFSE-stained PBMCs were added to the culture. The cultures were incubated for 5 days at 37°C in a humidified atmosphere of 5% CO_2_. At the end of co-culture, PBMCs were harvested for further cytometric analysis.

### Data Analyses

Data were analyzed using GraphPad Prism software version 7. The Shapiro-Wilk test was used as a normality test. One-way ANOVA with repeated measures and *post hoc* Tukey test was used to assess the effect of untreated and TI-treated ASCs on target cells and to compare contacting versus (vs) non-contacting co-cultures. The differences between ASC lines from healthy donors (HD/ASCs) and ASCs from SLE (SLE/ASCs) and SSc (SSc/ASCs) patients were analyzed using the Kruskal–Wallis and Dunn’s multiple comparison tests. Parametric (Pearson’s linear) and non-parametric (Spearman’s rank) correlation tests were used to assess an association between analyzed parameters. Probability values less than 0.05 were considered significant.

## Results

### Patients

The demographic and clinical data of the patients are shown in Table 1. The majority of patients presented low disease activity, and only 3 SLE and 2 SSc patients had active disease. All patients were ANA positive, and the majority of them had disease-specific autoantibody. However, SSc patients had higher ANA titer, while SLE patients were characterized by more diversified autoantibody profile and proteinuria. All patients were treated with immunosuppressive drugs, and none received biological therapy.

### Inhibition of T-Cell Proliferation by Adipose Tissue-Derived Mesenchymal Stem Cells

**FIGURE 1:**
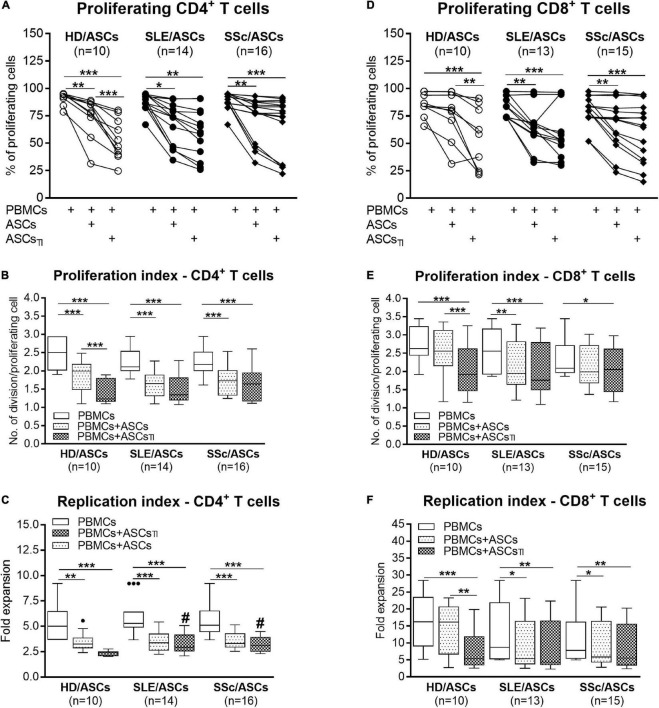
Inhibition of proliferation of CD4^+^ and CD8^+^ T cells by ASCs of healthy donors, SLE, and SSc patients in the co-cultures with peripheral blood mononuclear cells (PBMCs). PBMCs obtained from 9 healthy blood volunteers were stimulated with PHA and cultured alone (PBMCs) or co-cultured for 5 days with untreated (ASCs) or cytokine-licensed (ASCs_*TI*_) ASCs from 5 healthy donors (HD/ASCs), 9 SLE (SLE/ASCs), and 10 SSc (SSc/ASCs) patients. The proliferation of CD4^+^ **(A–C)** and CD8^+^ **(D–F)** T cells was analyzed by flow cytometry. **(A,D)** Lines between points identify the same combination of co-cultured ASCs and PBMCs. **(B–F)** Results of the indicated number of experiments (*n*), expressed as the median (horizontal line) with interquartile range (IQR, box), lower and upper whiskers (data within 3/2 × IQR), and outliers (points) (Tukey’s box). *P < 0.05, **P < 0.01, ***P < 0.001 for intra-group comparisons; ^#^*P* < 0.05 for the groups of patients versus HD comparison.

**FIGURE 2:**
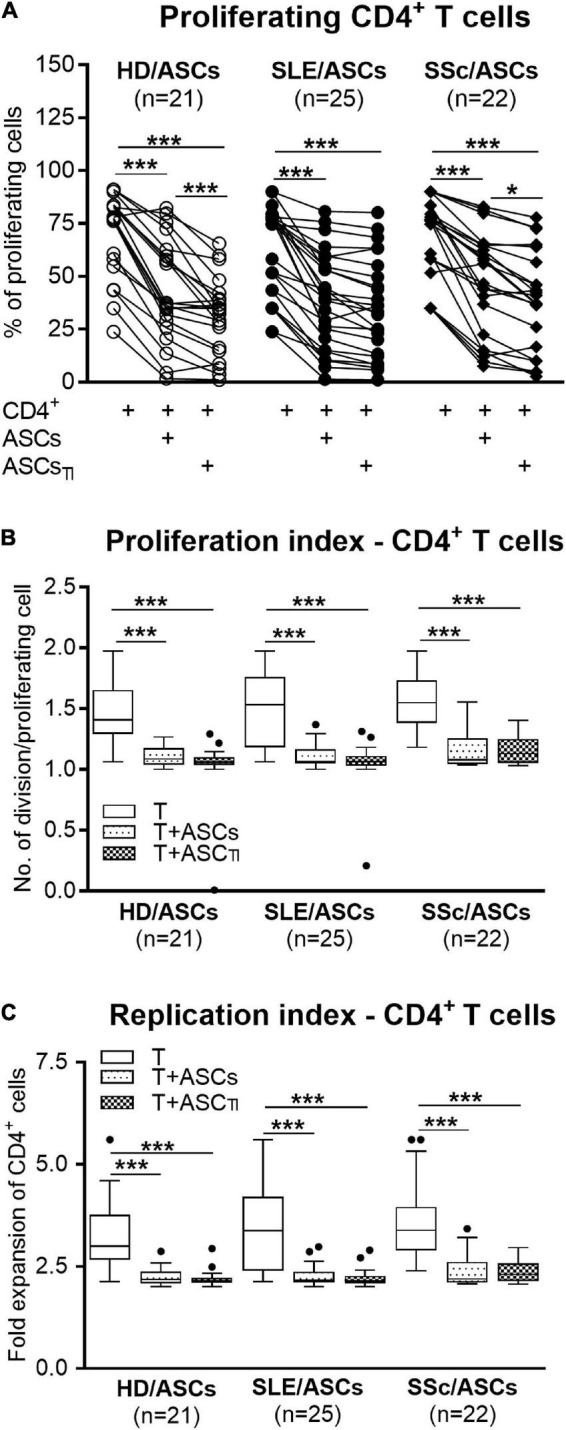
Inhibition of proliferation of purified CD4^+^ T-cells co-cultured with ASCs. CD4^+^ T-cells purified from PBMCs of 15 healthy blood volunteers were stimulated with anti-CD3/anti-CD28 antibodies and cultured alone (T) or co-cultured with ASCs or ASCs_*TI*_ obtained from 5 HD, 14 SLE, and 13 SSc patients. **(A)** After 5 days culture the proliferation of CD4^+^ T-cells was analyzed by flow cytometry. **(B)** Proliferation index and **(C)** replication index were calculated based on flow cytometry data. Other explanations as in Figure 1. *P < 0.05, **P < 0.01, ***P < 0.001 for intra-group comparisons.

Among PHA-treated PBMCs, the majority of CD4^+^ and CD8^+^ T cells proliferated (mean ± SEM = 89.07 ± 1.12 and 81.26 ± 1.89%, respectively), while the proportion of proliferating cells among anti-CD3/CD28 activated CD4^+^ T cells was lower (mean ± SEM = 66.99 ± 2.45%; *p* < 0.0001), yet the target cell donor-dependent variation was observed in both separate control cultures of activated PBMCs and CD4^+^ T cells (Figures 1A,D, 2A). In the presence of ASCs and ASCs_*TI*_, the number of proliferating T cells of both CD4^+^ and CD8^+^ subsets and the proliferation and replication indices decreased significantly in cultures containing PBMCs (Figure 1), and similar results were observed in cultures containing CD4^+^ T cells as the target cells (Figure 2). ASCs of patients exerted similar inhibitory effects as HD/ASCs (Figures 1A,B,D–F, 2A–C), except weaker, but statistically significant, diminution of CD4^+^ T-cell RI by ASCs_*TI*_ of SLE and SSc patients in PBMCs + ASCs co-cultures (Figure 1C). Cytokine-licensed HD/ASCs were more potent inhibitors of T-cell proliferation than untreated ones (Figures 1A,B,D–F, 2A), while inhibitory effects of ASCs and ASCs_*TI*_ of patients were comparable (Figures 1, 2). It should be mentioned that, in line with the previous observation (Kuca-Warnawin et al., 2021a), the degree of inhibition of proliferative response was also determined by the individual specificity of the donor of target cells (PBMCs or CD4^+^ T cells). For this reason, the activities of ASCs of healthy donors and tested patients were evaluated in the same experiments and using target cells obtained from the same donors (details are given in figure legends).

### Contribution of Cell-to-Cell Contact and Soluble Factors to the Anti-proliferative Action of Adipose Tissue-Derived Mesenchymal Stem Cells

**FIGURE 3:**
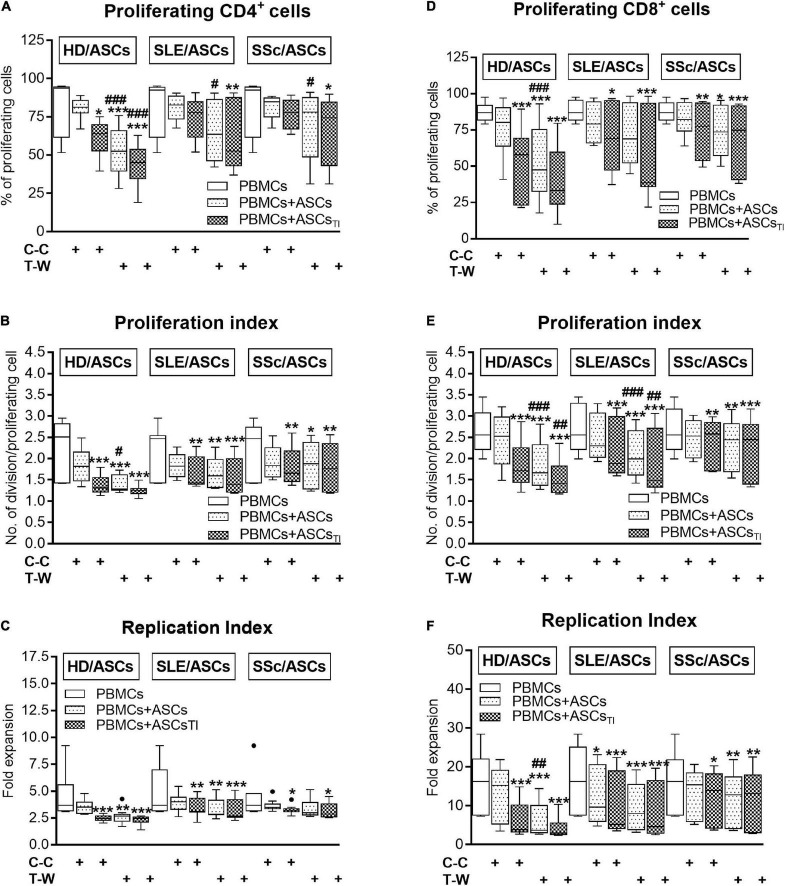
Comparison of ASCs inhibitory effects on **(A–C)** CD4^+^ and **(D–F)** CD8^+^ T-cell proliferation in cell contact and transwell co-cultures with PBMCs. PHA-activated PBMCs were cultured separately or co-cultured with ASCs or ASCs_*TI*_ in conditions allowing direct cell contact (C–C) or in a transwell system (T-W). Data, shown as the Tukey’s boxes, are the results of 9 experiments in which 5 HD/ASCs, 9 SLE/ASCs and 9 SSc/ASCs lines were co-cultured with PBMCs isolated from 7 healthy blood volunteers. *P < 0.05, **P < 0.01, and ***P < 0.001 for comparison of cell co-cultures vs. control separate cultures. ^#^*P* < 0.05, ^##^*P* < 0.01, and ^###^*P* < 0.001 for comparison of C-C vs. T-W co-cultures. Other explanations as in Figure 1.

**FIGURE 4:**
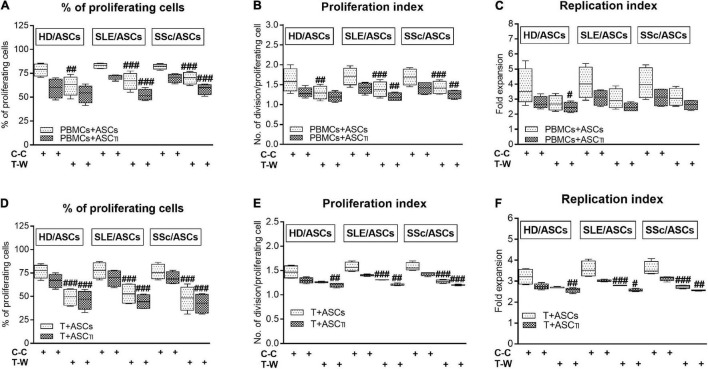
Comparison of ASCs inhibitory effects on CD4^+^ T-cell proliferation in cell contact and transwell co-cultures with PBMCs and purified CD4^+^ T cells isolated from the same healthy volunteers. Data are shown as the Tukey’s boxes and are results of 4 experiments in which 2 HD/ASCs, 2 SLE/ASCs, and 2 SSc/ASCs lines were co-cultured with PBMCs **(A–C)** or purified CD4^+^ T cells **(D–F)** isolated from the peripheral blood of 2 same healthy blood volunteers. Both PBMCs and purified CD4^+^ T cells were stimulated with PHA. ^#^*P* < 0.05,^ ##^*P* < 0.01, and ^###^*P* < 0.001 for comparison of C-C versus T-W co-cultures. Other explanations as in Figure 3.

In the co-cultures preventing (transwell, T-W) direct PBMCs and ASCs contact, untreated and, to a lesser extent, cytokine-licensed HD/ASCs reduced proliferation of CD4^+^ and CD8^+^ T cells (Figures 3A,B,D–F, respectively) more strongly than in co-cultures allowing cell-to-cell contact (C-C). For untreated HD/ASCs, these differences were observed in all measured proliferation indices, with the exception of replication index of CD4^+^ T cells (Figure 3C). Regarding TI-treated HD/ASCs, a similar tendency was observed, but significantly stronger reduction of the percentage of proliferating CD4^+^ cells and proliferation index of CD8^+^ cells in T-W than C-C conditions were only found (Figures 3A,E, respectively). Untreated ASCs of SLE and SSc patients exerted stronger inhibitory effect in T-W than C-C conditions merely on the percentage of proliferating CD4^+^ T cells (Figure 3A). In addition, both untreated and TI-treated SLE/ASCs diminished more potently the proliferation index of CD8^+^ T cells in T-W than C-C co-cultures (Figure 3E). These results underline the critical contribution of soluble factors to anti-proliferative capabilities of ASCs and suggest that cell-to-cell contact between ASCs and other cell types present in PBMCs pool may exercise control over anti-proliferative action of ASCs. To verify which cell type is engaged in such interaction, we performed additional experiments using target cells (PBMCs and T cells) obtained from the same healthy blood volunteers. As shown in Figure 4, untreated and/or cytokine-licensed HD/ASCs, SLE/ASCs, and SSc/ASCs exerted stronger anti-proliferative effect on T cells in T-W than C-C conditions, both when co-cultured with activated PBMCs and T cells (Figures 4A,B,D–F, respectively). In the co-cultures of ASCs with purified T cells, all tested proliferation indices were reduced more potently in T-W than C-C conditions (Figures 4D–F), while in analogous cultures containing PBMCs statistically significant differences were found only in the percentage of proliferating cells and proliferation index (**Figures 4A,B**), although similar tendency was noted for replication index (Figure 4C).

### Upregulation of Kynurenines and Prostaglandin E2, but Not Anti-inflammatory Cytokines, in the Co-cultures of Adipose Tissue-Derived Mesenchymal Stem Cells With Target Cells

**FIGURE 5:**
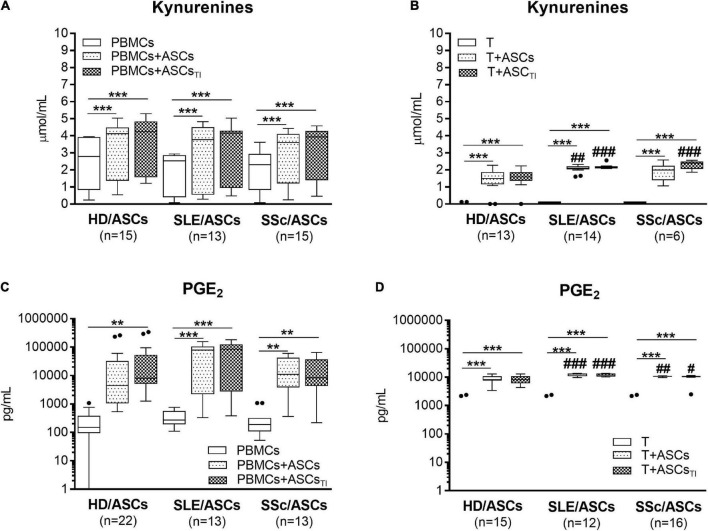
Upregulation of kynurenines and PGE_2_ synthesis in the co-cultures of ASCs with activated target cells. **(A,C)** PBMCs obtained from 13 healthy blood volunteers were stimulated with PHA and co-cultured in the same conditions as in Figure 1 with 5 HD/ASCs, 11 **(A)** or 7 **(C)** SLE/ASCs, and 10 **(A)** or 9 **(C)** SSc/ASCs. B,D: CD4^+^ T cells were purified from 12 healthy blood volunteers, stimulated with anti-CD3/anti-CD28 antibodies and co-cultured in the same conditions as in Figure 2 with 5 HD/ASCs, 8 **(B)** or 6 **(D)** SLE/ASCs, and 6 **(B)** or 8 **(D)** SSc/ASCs. The concentrations of kynurenines and PGE_2_ were measured in culture supernatants as described in the section “Materials and Methods.” Data are the results of the indicated number of experiments (*n*) and are shown as the Tukey’s boxes. *P < 0.05, **P < 0.01, ***P < 0.001 for comparisons of cell co-cultures versus separate control cultures; ^#^*P* < 0.05, ^##^*P* < 0.01, ^###^*P* < 0.001 for the groups of patients versus HD comparison. Other explanations as in Figures 1, 2.

**FIGURE 6:**
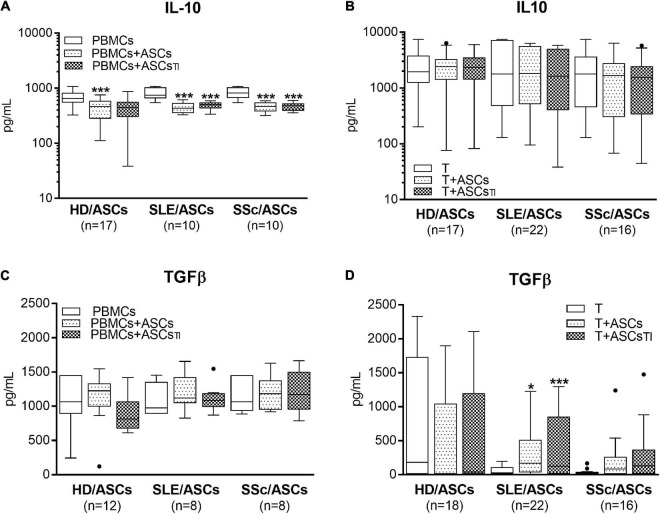
Concentrations of anti-inflammatory cytokines in the co-cultures of ASCs with activated target cells. **(A,C)** PBMCs obtained from 12 healthy blood volunteers were stimulated with PHA and co-cultured in the same conditions as in Figure 1 with 5 HD/ASCs, 9 **(A)** or 5 **(C)** SLE/ASCs, and 9 **(A)** or 5 **(C)** SSc/ASCs. **(B,D)** CD4^+^ T cells were purified from 9 healthy blood volunteers, stimulated with anti-CD3/anti-CD28 antibodies and co-cultured in the same conditions as in Figure 2 with 5 HD/ASCs, 11 **(B)** or 10 **(D)** SLE/ASCs, and 8 **(B)** or 9 **(D)** SSc/ASCs. The concentrations of TGFβ and IL-10 were measured in culture supernatants as described in the section “Materials and Methods.” Data are the results of the indicated number of experiments (*n*) and are shown as the Tukey’s boxes. *P < 0.05, **P < 0.01, ***P < 0.001 for comparisons of cell co-cultures versus separate control cultures. There were no statistically significant differences between co-cultures containing HD/ASCs versus ASCs of SLE and SSc patients. Other explanations as in Figures 1, 2.

Separately cultured activated PBMCs and CD4^+^ T cells produced similar amount of PGE_2_ (mean ± SEM = 312 ± 41 vs. 350.4 ± 134 pg/ml; *n* = 48 and *n* = 39, respectively), but moderate and negligible quantity of kynurenines (mean ± SEM = 2.08 ± 0.2 vs. 0.01 ± 0.005 mmol/ml, respectively; *n* = 33, *P* < 0.0001). Compared with these control cultures, significant increases of kynurenines and PGE_2_ concentrations were found in the co-cultures of activated PBMCs and CD4^+^ T cells with untreated and TI-treated ASCs of healthy donors as well as SLE and SSc patients (Figure 5). No statistically significant differences were found between co-cultures containing untreated and TI-treated ASCs. In the co-cultures of ASCs with purified CD4^+^ T cells, the production of kynurenines and PGE_2_ was significantly higher in the presence of ASCs from SLE and SSc patients than ASCs from healthy donors (Figures 5B,D), but it was similar in the co-cultures of ASCs with PBMCs (Figures 5A,C). Separately cultured activated PBMCs produced less IL-10 (mean ± SEM = 744 ± 36 vs. 1610 ± 244 pg/ml; *n* = 37 and 25, respectively; *P* = 0.003), but more TGFb (mean ± SEM = 1088 ± 64 vs. 297 ± 96 pg/ml; *n* = 28 and *n* = 44, respectively; *P* < 0.0001) than activated purified CD4^+^ T cells. In contrast to kynurenines and PGE_2_, the concentration of IL-10 did not change or even decrease in the co-cultures of ASCs with purified CD4^+^ T cell and PBMCs, respectively, and there was no difference between co-cultures containing HD/ASCs, SLE/ASCs, and SSc/ASCs (Figures 6A,B). The TGFβ levels fluctuated, but did not differ significantly between cultures, except the co-cultures of CD4^+^ T cells with SLE/ASCs and SSc/ASCs where significant upregulation or similar tendency was found, respectively (Figures 6C,D). These results suggested that kynurenines and PGE_2_, but not IL-10 and TGFβ, play a critical role in mediating the anti-proliferative effect of ASCs. This assumption was confirmed by findings showing an inverse correlation between the number of proliferating CD4^+^ and CD8^+^ T cells and kynurenines (*Rs* = –0.738, *P* < 0.0001 and *Rs* = –0.833, *P* < 0.001, respectively), and PGE_2_ (*Rs* = –0.645, *P* = 0.04 and *Rs* = –0.681, *P* = 0.03, respectively) found in co-cultures containing ASCs from tested patients (similar *Rs* values were found for TI-treated ASCs and other proliferation indices; data not shown). We have previously found the same associations between anti-proliferative effects of HD/ASCs and both kynurenines and PGE_2_ production (Kuca-Warnawin et al., 2021a).

### Contribution of Cell-to-Cell Contact and Soluble Factors to the Production of Kynurenines, Prostaglandin E2, and Anti-inflammatory Cytokines

**FIGURE 7:**
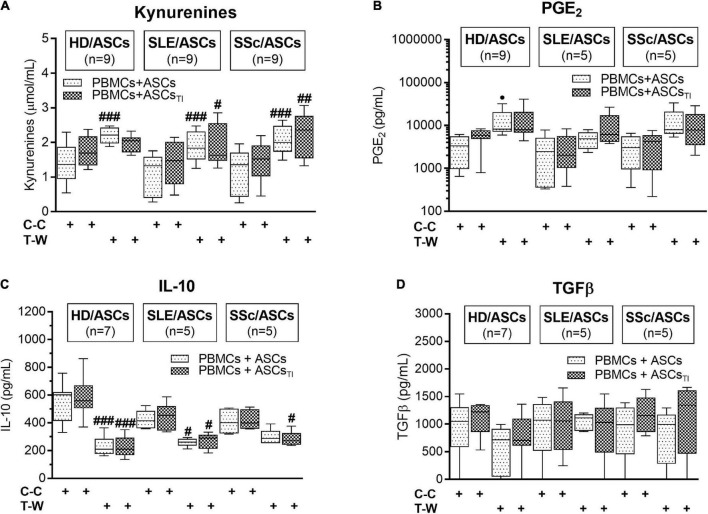
Concentrations of soluble mediators in cell contact and transwell co-cultures of ASCs with PBMCs: **(A)** Kynurenines, **(B)** PGE2, **(C)** IL-10, **(D)** TGFβ. The culture conditions and explanations as in Figure 3. Data are the results of indicated number of experiments *(n)* in which 5 HD/ASCs, 5 SLE/ASCs, and 5 SSc/ASCs were co-cultured with PBMCs isolated from 9 healthy blood volunteers and are shown as the Tukey’s boxes. ^#^*P* < 0.05, ^##^*P* < 0.01, and ^###^*P* < 0.001 for comparison of cell contact (C-C) vs. transwell (T-W) co-cultures.

As shown in Figure 7A, in the co-cultures of activated PBMCs with TI-treated and/or untreated ASCs of SLE and SSc patients, kynurenine production was significantly higher in T-W than C-C conditions. In the co-cultures of activated PBMCs with untreated HD/ASCs, the same effect in kynurenine production was observed. A similar tendency was noted in kynurenine production in co-cultures TI-treated HD/ASCs but the differences did not reach statistical significance. We observed a statistically non-significant trend toward higher PGE production in T-W cultures compared with C-C cultures in all tested ASCs (Figure 7B). In contrast, IL-10 production was higher in C-C than T-W cultures (Figure 7C), while TGFβ levels were similar in both conditions (Figure 7D). No significant differences in the concentrations of these soluble factors were found between co-cultures containing HD/ASCs, SLE/ASCs, and SSc/ASCs. These results show that similar to the anti-proliferative action of ASCs, the production of kynurenines and, to a lesser extent, the synthesis of PGE_2_ are out of control of cell-to-cell contact. In contrast, such cellular interaction seemed to be required for IL-10 production.

### Inhibitors of Kynurenine and Prostaglandin E2 Synthesis Abolish Anti-proliferative Effect of Adipose Tissue-Derived Mesenchymal Stem Cells

**FIGURE 8:**
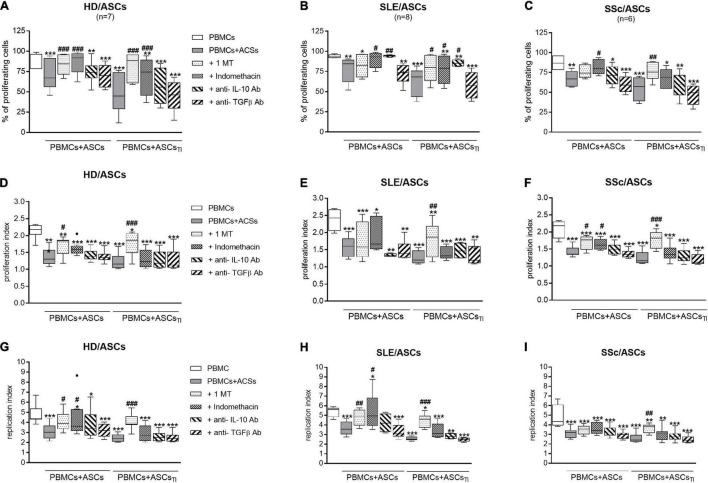
Selective inhibitors of kynurenines (1-MT) and PGE_2_ (indomethacin) synthesis counteract anti-proliferative impact of ASCs on CD4^+^ T-cells, while cytokine neutralization has negligible effect. Cell preparation and culture conditions as in Figure 1. Proliferation of CD4^+^ T-cells from PBMCs co-cultured with ASCs obtained from HD **(A,D,G)**, SLE **(B,E,H)** SSc **(C,F,I)** patients. The concentrations and time of cell treatment with 1-MT, indomethacin, and cytokine specific neutralizing antibodies are described in Materials and Methods. Data, shown as the Tukey’s boxes, are the results of indicated number of experiments (n) in which PBMCs obtained from 7 healthy blood volunteers were co-cultured with 3 HD/ASCs, 4 SLE/ASCs, and 5 SSc/ASCs. *P < 0.05, **P < 0.01, ***P < 0.001 for comparisons of cell co-cultures vs separate control cultures; ^#^*P* < 0.05, ^##^*P* < 0.01, ^###^*P* < 0.001 for comparison of inhibitor treated vs. untreated co-cultures. Other explanations as in Figure 1.

As shown in Figures 8A,D,G, 1-MT and indomethacin significantly abolished the anti-proliferative effect of untreated and TI-treated HD/ASCs. However, 1-MT reverted back all proliferative indices reduced by these cells, while indomethacin restored only the number of proliferating cells. Similar, but less pronounced, effects of these inhibitors were noted in co-cultures containing ASCs of patients. Although 1-MT restored all proliferation indices diminished by TI-treated ASCs of SLE (Figures 8B,E,H) and SSc (Figures 8C,F,I) patients, its effect on anti-proliferative action of untreated ASCs was weaker, and only the restoration of replication or proliferation indices was found, respectively. Indomethacin restored primarily the number of proliferating cells in the co-cultures of PBMCs with untreated and TI-treated SLE/ASCs (Figure 8B) as well as the number of proliferating cells and proliferation index in the co-cultures containing untreated SSc/ASCs (Figures 8C,F).

### Neutralization of Anti-inflammatory Cytokines Has Little Effect on Anti-proliferative Capacity of Adipose Tissue-Derived Mesenchymal Stem Cells

In contrast to the inhibitors of kynurenine and PGE_2_ synthesis, neutralization of TGFβ had no effect on the anti-proliferative activity of untreated and TI-treated HD/ASCs, SLE/ASCs, and SSc/ASCs (Figure 8). Interestingly, neutralization of IL-10 restored to a small, but statistically significant, extent the number of proliferating cells only in the co-cultures of PBMCs with untreated and TI-treated SLE/ASCs (Figure 8B). In addition, in the latter co-cultures, an inverse correlation between IL-10 levels and the number of proliferating CD4^+^ and CD8^+^ T cells (*R* = –0.684, *P* = 0.03 and *R* = –0.825, *P* = 0.003, respectively), proliferation index (*R* = –0.878, *P* = 0.0008 and *R* = –0.19, *P* = 0.04, respectively), and replication index (*R* = –0.631, *P* = 0.048 and *R* = –0.888, *P* = 0.006, respectively) were found (data not shown). Thus, IL-10 may contribute to anti-proliferative capability of SLE/ASCs, but its input to HD/ASCs and SSc/ASCs action seems to be negligible.

## Discussion

In searching for an effective and safe treatment for SLE and SSc patients, numerous research have focused on the therapeutic application of MSCs, known to possess immunomodulatory and regenerative potential. Because BM-MSCs of SLE and SSc patients have turned out to be functionally impaired (Maria et al., 2017; El-Jawhari et al., 2021; Tang et al., 2021), ASCs-based therapy seems to give a new hope for these patients (Rosa et al., 2021). However, the biological properties of SLE/ASCs and SSc/ASCs have not been thoroughly described yet. As for SSc/ASCs, conflicting results are currently available, since some authors provided evidence showing normal phenotype, proliferation rate, differentiation potential, pro-angiogenic, and anti-fibrotic properties (Capelli et al., 2017; Griffin et al., 2017; Velier et al., 2019), while others reported opposite results (Di Benedetto et al., 2019; Lee et al., 2019; Magalon et al., 2019). Moreover, only few papers have investigated the immunoregulatory activity of SSc/ASCs (Capelli et al., 2017; Kuca-Warnawin et al., 2019, 2020). In SLE, after finding senescence of BM-MSCs and dysfunction of these cells in both regenerative and immunoregulatory capabilities, studies have focused on the therapeutic application of allogeneic MSCs of various origins (Cheng et al., 2019). Although in the murine lupus models such treatments, including transplantation of human ASCs, are usually beneficial (Park et al., 2015; He et al., 2016; Wei et al., 2019), there is inconsistency in the effectiveness of MSCs treatment of SLE patients (Deng et al., 2017; Sharma et al., 2017; Zhou et al., 2020), and in clinical trials, mostly allogeneic BM-MSC or umbilical cord MSCs (UC-MSCs), but not ASCs, are used (Zhou et al., 2020). Therefore, before the therapeutic application of SLE/ASCs and SSc/ASCs in the autologous context, it is important to definitely clear up whether these cells possess similar beneficial properties as HD/ASCs. We have previously shown decreased basal levels of MSCs-specific CD90 marker and intracellular adhesion molecule (ICAM)-1 on ASCs of SLE and SSc patients, some changes in the secretory activity of these cells that may modify their biological functions, but their normal capability to regulate the expression of activation markers on allogeneic T cells (Kuca-Warnawin et al., 2019, 2020). In this study, we evaluated the anti-proliferative potential of ASCs, isolated from disease unaffected abdominal subcutaneous adipose tissue of SLE and SSc patients, toward allogeneic T cells, their mode (direct vs indirect) of action, and contribution of several known mediators to this immunoregulatory function of ASCs.

First, we have demonstrated that ASCs of SLE and SSc patients exert a similar inhibitory effect to HD/ASCs on the number of proliferating CD8^+^ and/or CD4^+^ T-cells, proliferation index, and replication index of CD8^+^ and/or CD4^+^ T-cells, irrespective of whether co-cultured with activated PBMCs or purified CD4^+^ T-cells (Figures 1, 2, respectively). Thus, the present results suggest that the ASCs of patients retain the normal capability to control T-cell proliferation *via* direct and indirect pathways. It should be mentioned that the inhibition of proliferation of the PHA-stimulated PBMCs by SSc/ASCs was also observed by other authors (Capelli et al., 2017), but in this article ASCs from only 3 SSc patients were evaluated. Consistent with other reports (Seo et al., 2019), we noticed that the anti-proliferative activity of HD/ASCs was enhanced by cytokine licensing, especially when co-cultured with activated PBMCs. In contrast, the cytokine pre-conditioning failed to increase such activity of ASCs of patients. By analogy to other rheumatic diseases (Kuca-Warnawin et al., 2021a), it is likely that an inability of SLE/ASCs and SSc/ASCs to intensify this immunoregulatory function upon TI-treatment results from *in vivo* exposure of these cells to the chronic systemic inflammatory milieu. The above difference in sensitivity of HD/ASCs versus SLE/ASCs and SSc/ASCs may partly explain slightly, but statistically significant, weaker reduction of CD4^+^ T-cell replication index by ASCs_*TI*_ of these patients co-cultured with activated PBMCs, as compared with HD/ASCs_*TI*_ (Figure 1C). It is known that upon TI-treatment, ASCs upregulate the expression of adhesion molecules and the production of soluble immunoregulatory mediators (Seo et al., 2019). We previously reported that TI licensing of ASCs from SLE and SSc patients resulted in an elevation of ICAM1/VCAM-1 expression to the levels similar to HD/ASCs (Kuca-Warnawin et al., 2019), while the present results show similar changes in the synthesis of immunomodulatory mediators (Figures 5, 6). Therefore, the mechanism(s) responsible for the incapability of ASCs of patients to enhance anti-proliferative activity remain unclear. However, this also implies that to efficiently control T-cell proliferation, ASCs of SLE and SSc patients do not rather require preconditioning with pro-inflammatory cytokines.

Consistent with numerous previous reports (Nasef et al., 2007; DelaRosa et al., 2009; Najar et al., 2010; Menta et al., 2014; Kuca-Warnawin et al., 2021a,b), we confirmed that in co-cultures with activated PBMCs, the anti-proliferative action of ASCs, both obtained from HD and SLE and SSc patients, toward CD4^+^ and CD8^+^ T cells, is critically dependent on soluble factors (Figure 3). Interestingly, we found that in a condition allowing cell-to-cell contact between ASCs and PBMCs, the inhibition of T-cell proliferation was weaker than that in the transwell system, suggesting a contribution of cellular interactions in the limitation of anti-proliferative action of ASCs. Regarding HD/ASCs, especially untreated ones, these differences were seen in almost all measured proliferation indices and thus were more evident than for ASCs of tested patients. In the case of the latter cells, stronger inhibition of T-cell proliferation concerned primarily the number of proliferating cells. Additional experiments, performed with two types of target cells, i.e., PBMCs and purified T cells, isolated from the same healthy blood volunteers revealed that, irrespective of target cell types and ASCs origin, the anti-proliferative action of ASCs was significantly weaker in cell contacting co-cultures than transwell system (Figure 4). Nevertheless, in the co-cultures of ASCs with PBMCs, such differences were in the number of proliferating cells and proliferation, but not replication, index, while in T-cell co-cultures, they were seen in all tested proliferation parameters. Therefore, it is tempting to speculate that interactions of ASCs with activated T cells *via* soluble factors deliver stronger anti-proliferative signals, while direct cell-to-cell contact may somehow restrict and thus control the anti-proliferative impact of ASCs. Other authors have also reported that if UC-MSCs make contact with lymphocytes isolated from patients with autoimmune diseases, their immunomodulatory function is hampered. To avoid MSCs contact with patient immune cells, they recommend the use of a microencapsulation technology for therapeutic transplantation of these cells (Alunno et al., 2018). There are several possible explanations for this finding. First, other authors demonstrated that in co-cultures, a part of activated T cells is bound to ASCs and kept in an activated, proliferative state (Quaedackers et al., 2009). Second, the low expression level of surface receptors mediating the immunosuppressive action of MSCs, e.g., CD90 and ICAM-1, may affect the anti-proliferative properties of these cells (Campioni et al., 2009; Liu et al., 2020). Although we previously found decreased basal expression of these receptors on ASCs of SLE and SSc patients, upon TI-treatment this defect normalized (Kuca-Warnawin et al., 2019). Moreover, a stronger reduction of T-cell proliferation was presently observed also in co-cultures containing HD/ASCs. Thus, decreased expression of these receptors is rather unlikely cause. Third, weaker control of anti-proliferative ASCs capabilities in co-cultures with PBMCs than T cells might be a net effect of multidirectional interactions between ASCs and different types of cells, e.g., monocytes, B lymphocytes, and NK cells, present in PBMCs pool that are able to deliver not only inhibitory (Cutler et al., 2010; Wang et al., 2010) but also pro-survival signals (Quaedackers et al., 2009; Ji et al., 2012; Healy et al., 2015; Zidan et al., 2020). And finally, the present results showing significantly higher concentrations of kynurenines and, to a lesser extent, PGE_2_ in transwell than cell contacting co-cultures of ASCs with PBMCs (Figure 7) provide another more probable clarification. It seems that the concentration of kynurenines is the most critical, because in PBMCs pool B and NK cells are more sensitive than T cells to tryptophan metabolites, especially up to the third day of *in vitro* co-culture with IDO-expressing dendritic cells (DC) (Terness et al., 2002). Thus, in our ASCs + PBMCs, co-cultures allowing cell-to-cell contact kynurenines, originating from ASCs, DC, and monocytes, are at first consumed by B and NK cells, while in ASCs + T cells, only T cells are exposed to them. In the transwell system, ASCs-derived kynurenines have to diffuse to reach T cells, while in the case of PBMCs, B cells, NK cells, and T cells are at first exposed to kynurenines produced by DC and monocytes than to kynurenines released from ASCs.

Because MSCs are known to perform their immunosuppressive function through the release of numerous mediators (Fan et al., 2020), we focused on the role of not only kynurenines and PGE_2_ but also SLE- and SSc-related cytokines, IL-10 and TGFβ, respectively. All these soluble factors have been proven to mediate MSCs immunomodulatory capacity (Nasef et al., 2007; Li et al., 2014; Fan et al., 2020). Moreover, both IL-10 and TGFβ possess immunosuppressive function and maintain a functional pool of regulatory T cells (Treg) (Liu et al., 2008; Guo, 2016). Besides, both cytokines contribute to disease pathology, IL-10 mostly *via* supporting hyperactivity of B cells and autoantibody production in SLE (Peng et al., 2013), while TGFβ is a potent inducer of fibrosis in SSc (Brown and O’Reilly, 2018), having a more complex role. Consistent with the above and our previous results (Kuca-Warnawin et al., 2021a,b), we recently confirmed significant upregulation of kynurenines and PGE_2_ levels in the co-cultures of ASCs with both PBMCs and purified CD4^+^ T cells (Figure 5), dependent mostly on soluble factors (Figures 7A,B). Compared with HD/ASCs, the concentrations of both mediators in the presence of ASCs of patients were similar or even higher, when PBMCs or T cells were used as the target cells, respectively. Thus, similar to preserved anti-proliferative capability, SLE/ASCs and SSc/ASCs turned out to be efficient producers of kynurenines and PGE_2_. In addition, an inverse correlation between the proliferation response of T cells and the concentrations of both kynurenines and PGE_2_ in the co-cultures of PBMCs with ASCs of patients was presently found, confirming the previous observation concerning HD/ASCs (Kuca-Warnawin et al., 2021a). In contrast, in the same co-culture conditions, there was no evident upregulation of either IL-10 or TGFβ (Figure 6). As for IL-10, significant downregulation of this cytokine level was found in all PBMCs + ASCs co-cultures. Because IL-10 is produced not only by ASCs but also by most types of leukocytes, especially over-activated T- and B-cells (Geginat et al., 2019), it is likely that this event, observed also by other authors (Özdemir et al., 2019), reflects the inhibitory effects of ASCs on lymphocyte activation, i.e., proliferation and cytokine production.

Therefore, the present results point out to kynurenines and PGE_2_ as crucial mediators of the anti-proliferative capability of ASCs originating from healthy donors as well as SLE and SSc patients. These observations are consistent with reports that have recognized kynurenine pathway as the most critical for ASCs inhibition of T-cell proliferation in humans (DelaRosa et al., 2009; Menta et al., 2014). Kynurenines are generated through catabolic degradation of the essential amino acid tryptophan (Trp) by indoleamine 2,3-dioxygenase (IDO). Local upregulation of kynurenine levels with concomitant deprivation of Trp suppresses T-cell proliferation and survival (King and Thomas, 2007; Munn and Mellor, 2013). Prostaglandin E_2_, the most abundant prostanoid found in the human body, has many important biological functions, including immunosuppressive role in T-cell immunity (Sreeramkumar et al., 2012), and numerous reports have documented PGE_2_ involvement in the inhibition of T-cell proliferation by MSCs of different origins (Cutler et al., 2010; Najar et al., 2010; Wang et al., 2010; Yañez et al., 2010). The prostanoids are synthesized from arachidonic acid processed by cyclooxygenase (COX) enzymes—constitutive COX-1 and inducible COX2, that generate PGG2, further reduced and converted to different types of prostaglandins, including PGE_2_ (Simmons et al., 2000; Sreeramkumar et al., 2012). Non-steroidal anti-inflammatory drugs, including indomethacin, are non-selective inhibitors of COX enzymes and thus inhibitors of prostaglandin synthesis (Simmons et al., 2000), while the kynurenine pathway can be blocked by Trp analog, 1-methyl-tryptophan (1-MT) (Wirthgen et al., 2018). To finally confirm the involvement of analyzed soluble mediators to ASCs anti-proliferative capability, we performed blocking experiments using 1-MT, indomethacin, and neutralizing antibodies specific to IL-10 and TGFβ (Figure 8). Although the results of blocking experiments supported the critical role of kynurenines and PGE_2_, they also demonstrated an unequal contribution of these mediators to ASCs function. Importantly, in mediating the suppressive effects of untreated and cytokine-licensed HD/ASCs, the kynurenine pathway reduced the number of proliferating T cells, the number of their division, and finally fold expansion, while PGE_2_ exerted inhibitory effect mostly on the number of proliferating cells. As for ASCs of SLE and SSc patients, similar differences were noted, but the effects of applied inhibitors were less pronounced, suggesting implication of other soluble factors. Importantly, in the case of SLE/ASCs neutralization of IL-10 partly, but significantly, restored proliferation of T cells. Moreover, IL-10 levels inversely correlated with T-cell proliferation only in co-cultures with SLE/ASCs. Therefore, our findings identified IL-10 as an additional mediator of anti-proliferative capabilities of SLE/ASCs, adding new evidence of this cytokine contribution to immunoregulatory mechanisms known to be mediated by regulatory lymphocytes (Treg, Breg) (Geginat et al., 2019). Interestingly, human ASCs were reported to induce the expansion of IL-10-producing Breg cells and significantly ameliorated autoimmunity in a murine model of SLE (Park et al., 2015), showing the beneficial role of this mechanism *in vivo*.

## Conclusion

In summary, we report that ASCs obtained from SLE or SSc patients have preserved the capability to sufficiently inhibit the proliferation of two main T-cell subsets (CD4^+^ and CD8^+^) through direct and indirect ways. In this ASCs action, the soluble mediators, most of all kynurenines and PGE_2_, deliver strong anti-proliferative signals, while direct cell-to-cell interactions seem to convey also restriction signals. Moreover, the kynurenine pathway mediates the anti-proliferative effect of ASCs by inhibiting both the number of proliferating cells, the number of their division, and fold expansion, whereas the PGE_2_ has a smaller contribution as it reduces mainly the number of proliferating cells. The TGFβ does not seem to play any role, but the IL-10 seems to mediate to some extent the anti-proliferative action of ASCs of only SLE patients. As there were no crucial differences between ASCs from healthy donors and tested patients, these cells may represent an optional source for the therapy of SLE and SSc patients.

## Data Availability Statement

The original contributions presented in the study are included in the article/supplementary material, further inquiries can be directed to the corresponding author.

## Ethics Statement

The studies involving human participants were reviewed and approved by Ethics Committee of the National Institute of Geriatrics, Rheumatology and Rehabilitation, Warsaw, Poland. The patients/participants provided their written informed consent to participate in this study.

## Author Contributions

EK-W designed the experiments, performed the flow cytometry experiments, and analyzed data. PS and MO helped with recruiting patients, and acquired and analyzed the clinical data. EK wrote the manuscript. All authors have read and approved the submitted version.

## Conflict of Interest

The authors declare that the research was conducted in the absence of any commercial or financial relationships that could be construed as a potential conflict of interest.

## Publisher’s Note

All claims expressed in this article are solely those of the authors and do not necessarily represent those of their affiliated organizations, or those of the publisher, the editors and the reviewers. Any product that may be evaluated in this article, or claim that may be made by its manufacturer, is not guaranteed or endorsed by the publisher.
